# Phenotypic screening in zebrafish larvae identifies promising cyanobacterial strains and pheophorbide a as insulin mimetics

**DOI:** 10.1038/s41598-024-83986-0

**Published:** 2024-12-30

**Authors:** Tiago Ribeiro, Mariana Reis, Vitor Vasconcelos, Ralph Urbatzka

**Affiliations:** 1 Interdisciplinary Centre of Marine and Environmental Research (CIIMAR/CIMAR), University of Porto, Avenida General Norton de Matos, s/n, Matosinhos, 4450-208 Portugal; 2 Faculty of Sciences, University of Porto, Rua do Campo Alegre, Porto, 1021, 4169-007 Portugal

**Keywords:** Diabetes, Zebrafish, Glucose, Metabolomics, Pheophorbide a, Cyanobacteria, Phenotypic screening, Diabetes

## Abstract

Diabetes is a pandemic disease that causes the loss of control of glucose regulation in the organism, in consequence of dysfunction of insulin production or functionality. In this work, the antidiabetic bioactivity of 182 fractions from 19 cyanobacteria strains derived from the LEGE Culture Collection were analysed using the 2-NBDG assay in zebrafish larvae. From this initial screening, two fractions (57 (**06104_D**) and 107 (**03283_B**)) were identified as promising insulin mimetics. These were further characterized by measuring glucose levels in whole larvae, the expression of glucose transporters (GLUT 1–3) using western blot, and the mRNA expression levels of the *glut2*, *pepck*, and *insa* genes using real-time qPCR. Both fractions showed a decrease in free glucose levels. Furthermore, exposure to fraction 06104_D decreased GLUT1 and increased *insa* mRNA levels. The chemical composition of these fractions was determined using LC-HRESIMS/MS and compared to inactive fractions of the same polarity in order to identify the unique bioactive molecules. The molecular networks constructed using the GNPS platform revealed that fraction 06104_D contained mass clusters primarily composed of chlorins, lipids, and terpenoids, while fraction 03283_B contained xanthophylls, peptides, and terpenoids. To correlate the observed activity with the chemical composition of fraction 06104_D, pheophorbide a was chosen as a representative of chlorophyll derivatives. Exposure to zebrafish larvae at 10 and 20 µM confirmed the increased glucose uptake on the 2-NBDG assay. These findings highlight the bioactivity of chlorophyll derivatives as insulin mimetic compounds, as well as cyanobacteria as a source of potential therapeutic diabetes applications.

**Supplementary Information:**

The online version contains supplementary material available at 10.1038/s41598-024-83986-0.

## Introduction

Diabetes is a worldwide pandemic disease affecting more than 400 million people^1^. This ailment can be divided in two major types: diabetes type I, based on insulin deficiency; and diabetes type II, based on insulin resistance. These two pathologies cause different physiological consequences, as cognitive impairments or mitochondrial dysfunction. The overlap of both conditions of insulin deficiency and resistance, confined in the brain, can lead to a status of neurodegeneration. The development of this condition, in consequence of insulin deregulation, can lead to a clinical status very similar to the Alzheimer’s disease, also known as diabetes type III^2^.

The treatment of diabetes is mostly based on a daily control of glucose levels and administration of insulin when the levels of glucose are high^3^. Other drugs inhibit the release of glucagon, the molecule responsible to increase glucose levels, like Tirzepatide^4^, or sodium-glucose co transporters-2, which stimulates elimination of glucose by the kidneys^5,^ .

The control of glucose levels in the organism is a very complex mechanism. When glucose levels are low, the pancreas secretes glucagon, a peptide hormone that stimulates glycogenolysis, converting glycogen into glucose^6^. Insulin is synthesized by the β-cells of the pancreatic islets. This protein controls the glucose levels in the organism by regulating the activity of the glucose transporters (GLUTs), especially GLUT2 and GLUT4, present on the liver and skeletal muscle/adipose tissue, respectively^7^. GLUTs have many different roles, including the transport of glucose and other molecules, especially other carbohydrates like fructose, as well as specific tissue distribution^8^.

Despite the exisiting pharmacotherapies for diabetes, novel solutions are still needed. In particular, insulin mimeting compounds could be helpful in the context of insulin resistance. Natural products are a great source of bioactive compounds, and could provide molecules with antidiabetic potential, either as the basis for structural improvements by medicinal chemistry, or as new drugs in the future^9^. Previous research highlighted some of the potential in natural resources. The oral administration of extracts from the plant *Ceriops decandra*, a type of mangrove, at a concentration of 120 mg/kg, reduced the diabetic condition of alloxan-induced diabetic rats after 30 days^10^. Extracts of the fungus *Nigrospora oryzae*, were also found to reduce fasting blood glucose levels in alloxan-induced diabetic mice^11^, . Octaphloretol, a phlorotannin isolated from the algae *Ishige foliacea*, demonstrated a strong reduction of the glucose levels in streptozotocin-induced diabetic mice, following oral administration at 100 mg/kg after a minimum of 30 min^12^. Phycocyanin, extracted from the cyanobacterium *Spirulina platensis* inhibited α-amylase and β-glucosidase in vitro with IC_50_values of 231.45 and 198.11 µg/mL, respectively^13^.

Given the potential of natural products for drug discovery, the aim of this work was explore cyanobacteria as a source of insulin mimetic compounds. CIIMAR (Interdisciplinary Centre of Marine and Environmental Research - Portugal) houses the Blue Biotechnology and Ecotoxicology Culture Collection (LEGE-CC, https://lege.ciimar.up.pt) a biodiversity resource with over 1000 cyanobacterial and microalgae strains collected from various ecosystems and countries around the world (e.g. Portugal, Brazil, Cape Verde, Mexico, Australia). Some of these have already demonstrated the ability to produce bioactive compounds with anti-cancer, anti-biofilm, or lipid-reducing properties^14,15^. Here, we conducted the first phenotypic screening approach on a library of 182 cyanobacterial fractions to find novel insulin mimetic compounds, using the 2-NBDG assay on zebrafish larvae, which measures the uptake of a fluorescent glucose analogue. Two promising fractions were identified and their biochemical mechanisms were further investigated by analysing whole body glucose levels, protein expression of glucose transporters and the mRNA expression of key glucose metabolism genes. An untargeted metabolomics approach was used to identify the bioactive molecules present in the promising fractions. The molecular networks were constructed with Global Natural Products Social Molecular Networking (GNPS) using two different methodologies. Multiple clusters of terpenoids, some lipid classes, and derivatives of chlorophyll were identified that might be connected to the observed insulin mimetic activity. Pheophorbide a, a pure chlorophyll derivative, showed anti-diabetic potential on zebrafish larvae. These results highlight the interest in chlorophyll derivatives as potential insulin mimetics, for further follow-up in the future.

## Results

### Glucose uptake screening on zebrafish larvae

**Fig. 1:**
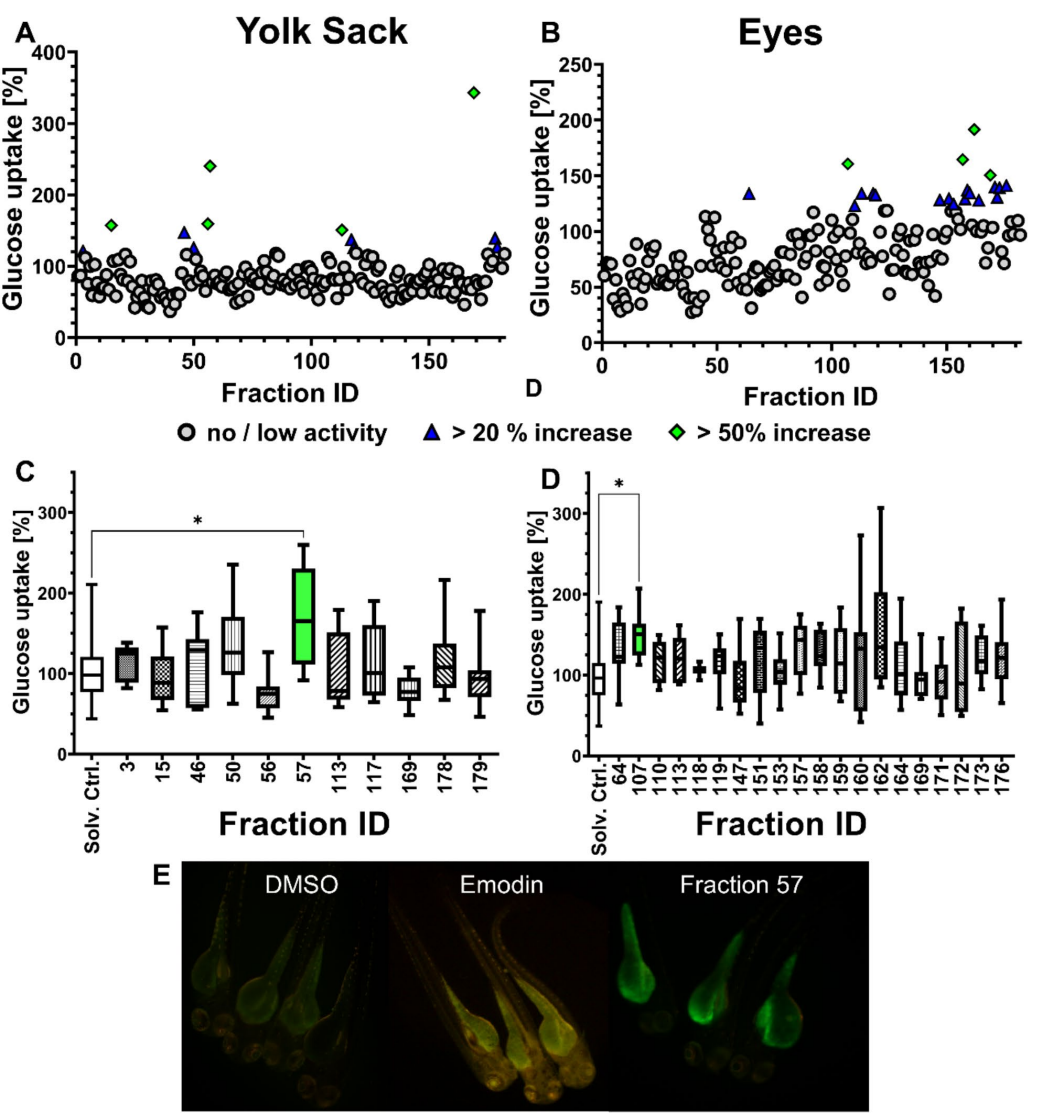
Screening for insulin mimetic activity in zebrafish larvae exposed to cyanobacterial fractions using the 2-NBDG assay. Percentage of glucose uptake relative to solvent control (DMSO 0.1%) was evaluated. 182 fractions were screened and fluorescence was quantified in the yolk sac (A) and in the eye (B). Fractions with an increase > 20% are shown in blue, and > 50% in green. Promising fractions from the initial screening were tested in an additional assay. Fractions marked in green increased significantly 2-NBDG uptake in the yolk sac (C) or eye (D). The data is represented as box-whisker plots from the fifth to 95th percentiles. Asterisks highlight significantly altered fluorescence intensities that indicate changes in 2-NBDG uptake (* *p* < 0.05), Kruskal-Wallis and Dunn’s test. E) Representative images from fluorescence microscopy: solvent control (DMSO, 0.1%); positive control (emodin, 10 µM) and fraction 57, 10 µg/mL.

19 cyanobacteria strains that were previously not explored for these bioactivities were extracted and fractionated. The resulting 182 fractions were tested at 10 µg/ml final concentration on the 2-NBDG assay on 3 – days post fertilization (dpf) zebrafish larvae (Fig. 1) to evaluate the increase of glucose uptake in vivo in the yolk sac and in the eyes. Promising fractions were defined as those that were non-toxic and had a 2-NBDG signal increase of more than 20% compared to control. The fluorescence quantification revealed a total of 29 promising fractions: 9 on the yolk sac (fractions 3, 15, 46, 50, 56, 57, 117, 178 and 179), 17 on the eye (fractions 64, 107, 110, 119, 147, 151, 153, 157, 158, 159, 160, 162, 164, 171, 172, 173 and 176) and 3 both on the yolk sac and eye (fractions 113, 118 and 169). Those fractions were tested again in additional, independent assays to confirm their bioactivity, and two positive hits were validated. Fraction 57, from the marine cyanobacteria *Nodosilinea nodulosa* LEGE 06104, fractionated with 60% Hexane/40% Ethyl Acetate (D), increased 2-NBDG uptake in the yolk sac, while fraction 107, from the freshwater cyanobacteria *Nodosilinea* sp. LEGE 03283, fractionated with 80% Hexane / 20% Ethyl Acetate (B), increased 2-NBDG uptake in the eye. From this point on, fraction 57 will be named 06104_D and fraction 107 as 03283_B.

## Glucose measurement

**Fig. 2:**
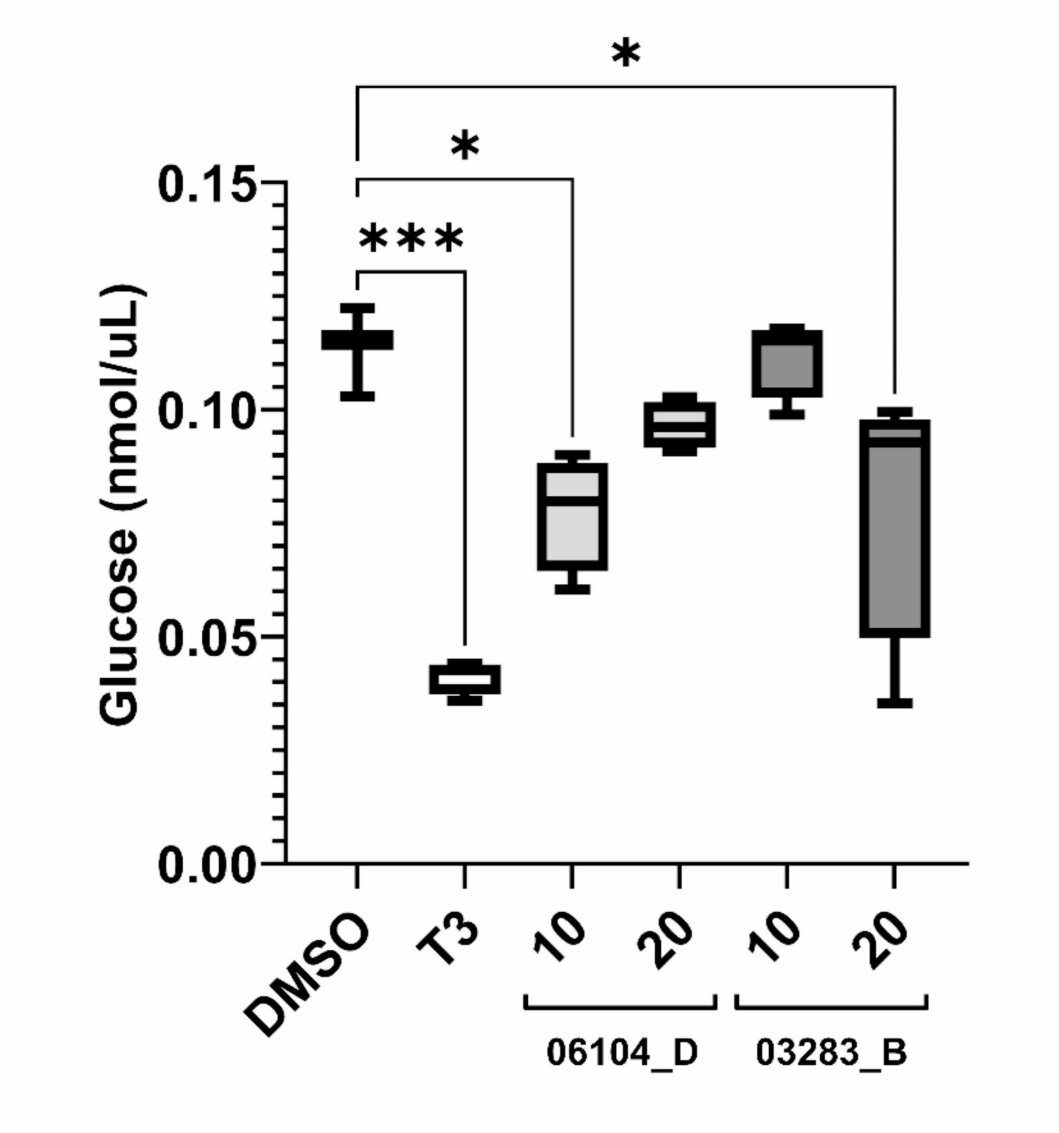
Glucose measurement on 4 dpf zebrafish embryos, exposed to Fraction 06104_D and 03283_B (at 10 µg/mL and 20 µg/mL), as well to the solvent control, DMSO (0.1%), and positive control, T3 (10 µM). Three replicates were used in two independent assays, *n* = 6. The data is represented as box-whisker plots from the fifth to 95th percentiles. Asterisks show significant differences vs. the solvent control (*** *p* < 0.001; ** *p* < 0.01; * *p* < 0.05), Kruskal-Wallis and Dunn’s test.

To verify the effect on the uptake of glucose, 4 dpf embryos were exposed for 24 h to and controls and positive fractions 06104_D and 03283_B, at 10 and 20 µg/ml final concentrations. Triiodothyronine (T3) at 10 µM was used as a positive control in this assay, since it is known to stimulate insulin production, increasing the uptake of glucose^16^. As expected, T3 reduced significant the free glucose levels (Fig. 2), as it increased the uptake of glucose and its subsequent consumption or conversion by the embryos. Fraction 03283_B showed a reduction of glucose levels at the highest concentration (20 µg/mL), while fraction 06104_D showed a decrease at the lower concentration (10 µg/mL). In contrary to expected, the higher concentration (20 µg/mL) of fraction 06104_D, didn’t show any statistical significant effect.; no toxic effects or lethality were observed during exposures. Taken together with the 2-NBDG assay results, these finding indicate that both fractions increased glucose uptake leading to their consumption/conversion by the zebrafish larvae.

## Phylogenetic analysis of the glucose transporters

**Fig. 3:**
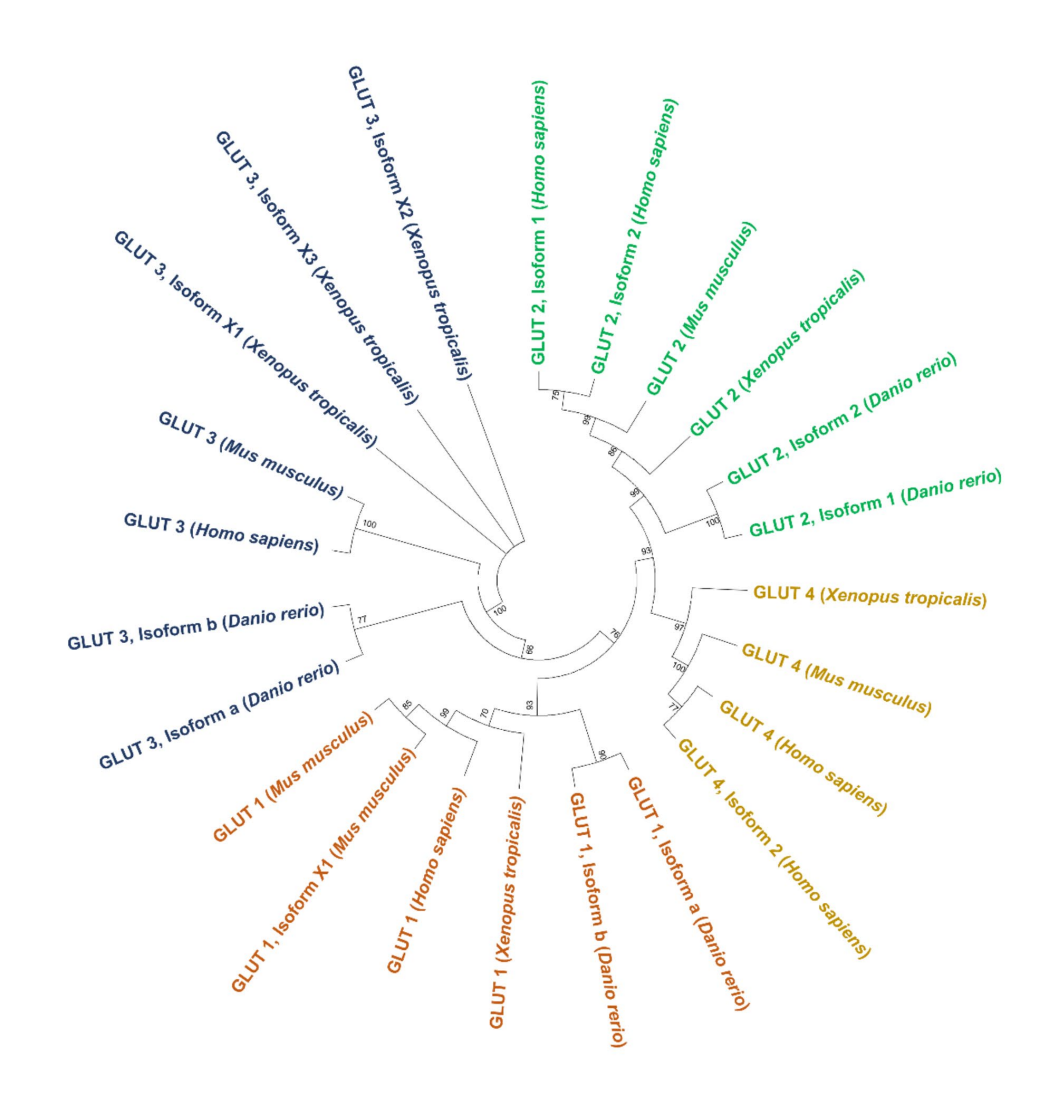
The evolutionary history of glucose transporters (GLUT 1–3) with the sequences of *Mus musculus*, *Homo sapiens* and *Xenopus tropicalis* was inferred by using the Maximum Likelihood method based on the JTT matrix-based model. The tree with the highest log likelihood (−4071.74) is shown. The percentage of trees in which the associated taxa clustered together is shown next to the branches. The analysis involved 23 amino acid sequences. All positions containing gaps and missing data were eliminated. There was a total of 260 positions in the final dataset. Evolutionary analyses were conducted in MEGA7 ^17^.

As the glucose transporters (GLUTs) are known for their importance in glucose uptake, the phylogenetic proximity of class I of major glucose transporters from zebrafish (GLUT 1–4) was analysed in comparison to *Mus musculus*, *Homo sapiens* and *Xenopus tropicalis*. As shown in Fig. 3, all the GLUTs cluster by their type between the four species. Glut1 of zebrafish clustered together with the other Glut1 sequences, similarly to Glut2 and Glut3. These observed sequence similarities between species suggest a conserved function and may allow a better comparison of the zebrafish results with other species as mice or human. However, for glucose transporter GLUT4, no genetic sequences for zebrafish were available in NCBI and Ensembl, indicating potential gene loss. Commercial antibodies for zebrafish GLUT4 are on the market, but it remains unclear of what they bind to.

## Glucose transporter expression by western blot

**Fig. 4:**
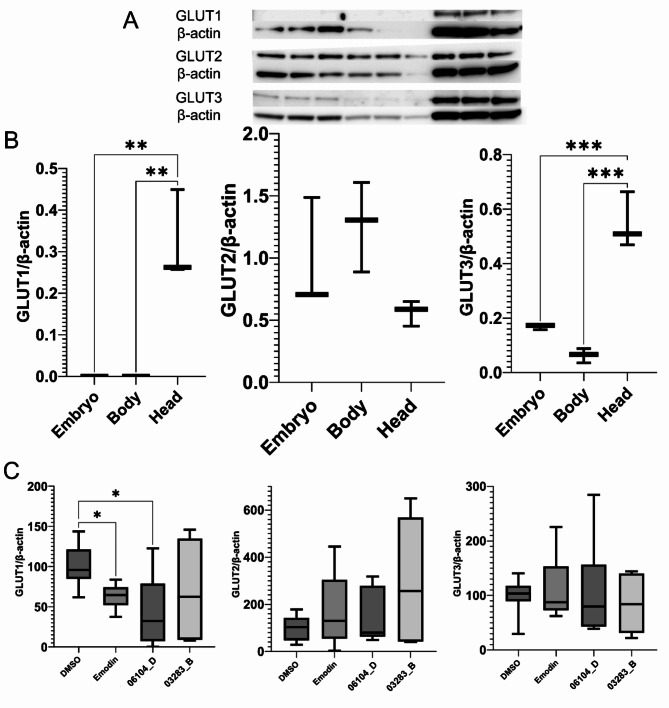
Western blot analysis of glucose transporters (GLUTs). A and B: GLUT expression from whole zebrafish embryos (3 dpf) and from head, and body, including the yolk sac. The expression of each GLUT and respective β-actin on the membrane is shown on A (top GLUT, bottom β-actin). B: quantification of the relative expression of each GLUT normalized by β-actin. C: GLUT protein level from zebrafish embryos (3 dpf) normalized to β-actin, after exposure to fractions 06104_D and 03283_B at 10 µg/ml and Emodin at 10 µM, using DMSO (0.1%) as solvent control. Three replicates consisting of a pool of 16 larvae each were used per condition in two independent assays (*n* = 6). The data is represented as box-whisker plots from the fifth to 95th percentiles, and asterisks highlight significant differences vs. the solvent control (*** *p* < 0.001; ** *p* < 0.01; * *p* < 0.05), One-Way ANOVA and Dunnett’s posthoc test.

The expression of glucose transporters was assessed by western blot on the head and body of zebrafish embryos. According to the literature, glucose transporters in humans should exhibit a differential expression, with GLUT 1 and 3 being more expressed on the head and GLUT 2 having a more general distribution throughout the organism^7^. As shown in Fig. 4 (A and B), a differential expression of GLUT 1 and GLUT3 was observed, with a higher expression in the head relative to the body or the entire embryo. As expected, GLUT2 did not have a differential expression between head and body of the zebrafish embryo.

As observed in the 2-NBDG screening, fractions 06104_D and 03283_B exhibited selective effects in the yolk sack and eye, respectively. To investigate whether these effects can be associated with specific glucose transporters, we evaluated the expression of GLUTs in zebrafish embryos after a 1-hour exposure to the fractions 06104_D and 03283_B at 10 µg/mL. Exposure to fraction 06104_D resulted in a lower protein level of GLUT1 compared to the solvent control (0.1% DMSO). This effect is similar to the one of the positive control emodin at 10 µM (Fig. 4C). No significant differences in the protein level of GLUT 2 and 3 were observed between the controls and the fractions 06104_D and 03283_B (Fig. 4C).

## mRNA expression

**Fig. 5:**
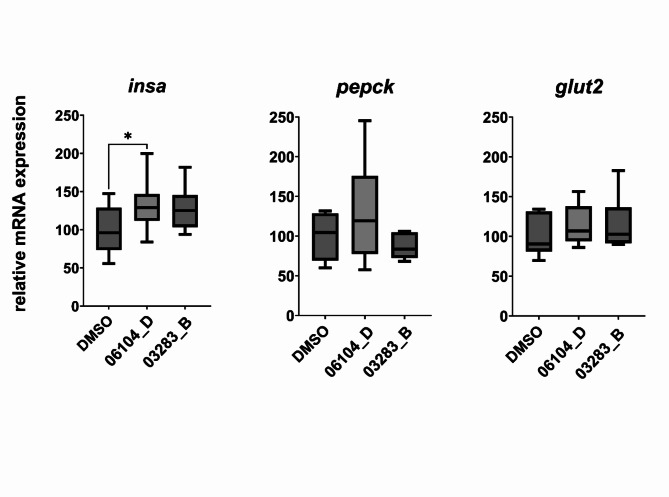
Relative mRNA expression of *insa*, *pepck* and *glut2* from zebrafish embryos (*Danio rerio*), after 24 h of exposure to solvent control (DMSO 0.1%) and fractions 06104_D and 03283_B at 10 µg/mL. Data are expressed as mean ± SD (*n* = 6 for each group), using box-whisker plots from the fifth to 95th percentiles. Asterisks highlight significantly altered mRNA expression (* *p* < 0.05), One-Way ANOVA and Dunnett’s posthoc test.

To assess the effect of fractions 06104_D and 03283_B at 10 µg/mL on mRNA expression of genes involved in glucose metabolism, three genes were selected: *glut2* - glucose transporter 2; *insa* - insulin isoform A (more functionally similar to the human insulin) and *pepck* - phosphoenolpyruvate carboxykinase, key enzyme on the gluconeogenesis pathway. Figure 5 summarizes the results of the target gene mRNA expression, normalized by two housekeeping genes, *rpl8*, ribosomal protein 8, and *ube2l3*, ubiquitin-conjugating enzyme E2L3. Fraction 06104_D increased significantly the *insa* mRNA (*p* < 0.05) suggesting that it may stimulate the production of insulin, necessary to control the levels of circulating glucose.

### Metabolite profile

**Fig. 6:**
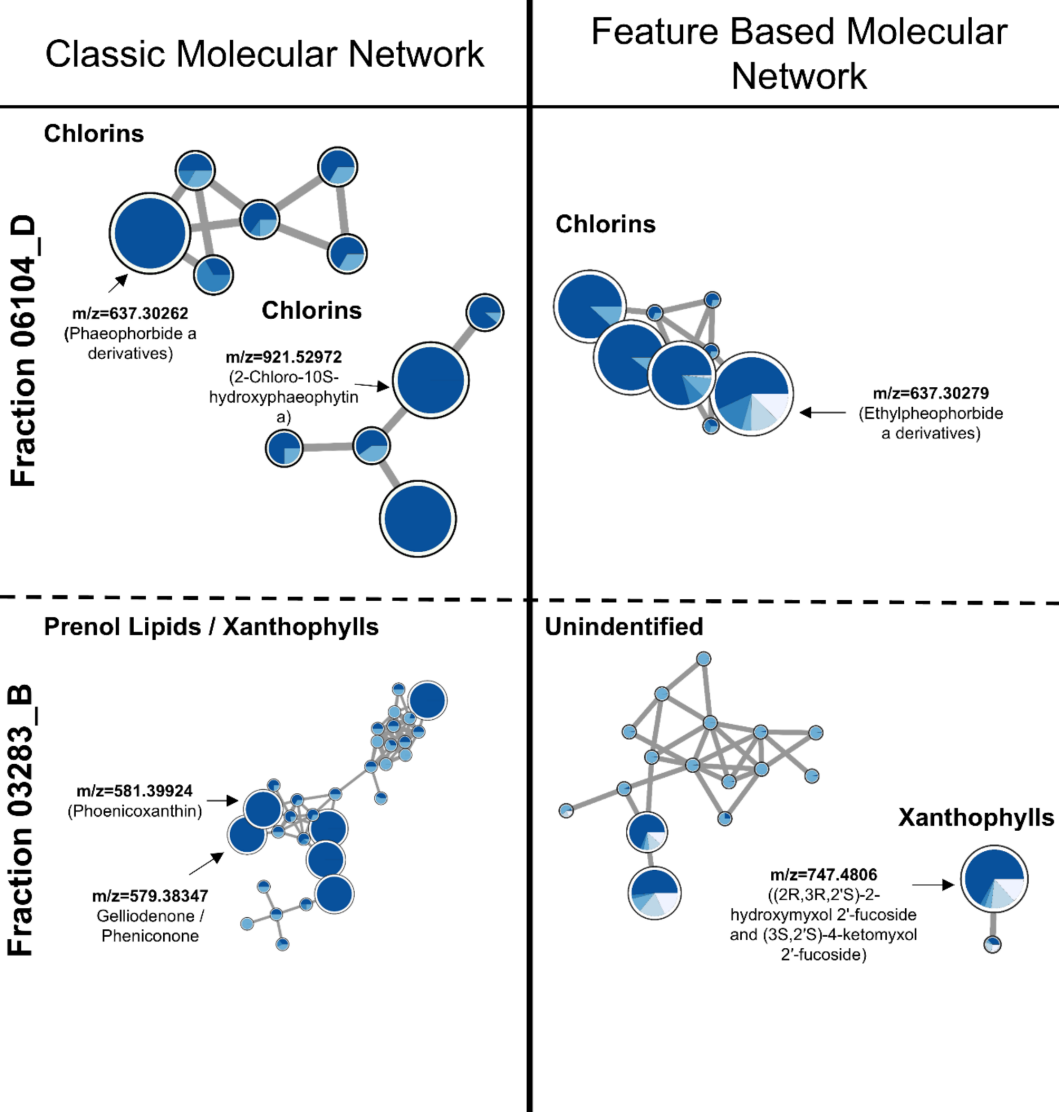
Cytoscape visualization of the main clusters of the final molecular network of fraction 06104_D (TOP) and 03283_B (BOTTOM), for each GNPS analysis approach. Compound families’ identifications were done using ClassyFire^18^. In the classic GNPS approach, larger nodes are masses that are unique to the bioactive fraction, whereas in the bioactivity-based molecular network approach, the size of the nodes is proportional to their significance to the observed bioactivity. Each color represents a fraction with the same solvent polarity from cyanobacteria strains belonging to the genus *Nodosilinea*. The dark blue color corresponds to the positive hits, fractions 06104_D and 03283_B, respectively, while the others are from non-active fractions. Networks derived from Cytoscape 3.10.0 23^19^.

The metabolite profile of fractions 06104_D and 03283_B was studied by high-resolution tandem mass spectrometry (LC-HR-ESI-LC-MS/MS). These data were compared to the metabolites of two inactive fractions that shared the same polarity and were members of the same genus, *Nodosilinea*. The classic GNPS molecular network was used to organize the chemical space in order to identify the mass features specific to the active fractions, whereas the bioactivity-based molecular networking was used to find masses that may be related to bioactivity. For fraction 06104_D, the classic GNPS approach identified 63 unique mass features. These could not be classified or dereplicated using the GNPS in silico tools. Therefore, a manual search of the MS1 databases Dictionary of Natural Products (DNP) and Natural Product Atlas (NPA) led to the tentative identification of 10 mass nodes (Table 1). The bioactive-based molecular network identified 51 unique mass nodes in fraction 06104_D, and the manual search on the databases retrieved 14 putative identifications (Supplementary Table 1). Three of the masses matched between the two types of metabolomics analysis namely for *m/z* 637.3028 [M + H]^+^ and *m/z* 921.5298 [M + H]^+^ putatively identified as cholorophyll a derivatives, and *m/z* 955.5787 [M + H]^+^, identified as aminoglycosides (Fig. 6).

For fraction 03283_B, the molecular network constructed using the classic GNPS workflow identified 52 unique mass nodes. Because no matches were found in the GNPS databases, these masses were manually searched in DNP and NPA, yielding 17 putative identifications (Supplementary Table 2). The bioactivity-based molecular network identified 9 masses, 7 of which were tentatively identified (Supplementary Table 3). As with fraction 06104_D, there was overlap between the nodes, namely *m/z* 569.3815 [M + H]^+^, putatively identified as integracin C. Nonetheless, fraction 03283_B revealed the presence of distinct nodes in clusters of xanthophylls such as phoenicoxanthin or 2-hydroxytorularhodin phoenicoxanthin (Fig. 6), peptides (e.g., amphimedoside or acremolides), and terpenoids, among others.

**Table 1:** Classic GNPS molecular networking of fraction 06104_D. Manual annotation of unique mass nodes was retrieved from natural product Atlas (NPA) and Dictionary of Natural products (DNP) databases, based on the percursor ion (*m/z*) and calculated molecular formula within a mass error of ± 5 ppm.

		Dicitionary of Natural Products				Natural Product Atlas			
m/z	Retention Time (min)	Molecular Formula	Mass error (ppm)	Tentative Identification	ClassyFire Class Annotation	Molecular Formula	Mass error (ppm)	Tentative Identification	ClassyFire Class Annotation
289.1537	9.72	C_16_H_20_N_2_O_3_	5	10 structures					
447.3833	12.59	C_29_H_50_O_3_	1	87 structures					
637.3026	12.07	C_37_H_40_N_4_O_6_	0	13^2^-Hydroxy-17,17^1^-dimethylphaeophorbide a and Ethyl 10-hydroxyphaeophorbide a	chlorins	C_37_H_40_N_4_O_6_	0	(13^2^S, 17 S, 18 S)−13^2^-hydroxy-20-chloro-ethylpheophorbide a	chlorins
815.5468	15.93	C_51_H_74_O_8_	0	ganoleuconin P and ganosinensin B.	Triterpenoids	C_51_H_74_O_8_	1	ganoleuconin P and ganosinensin C	triterpenoids
921.5297	15.23	C_55_H_73_ClN_4_O_6_	0	2-Chloro-10 S-hydroxyphaeophytin a	Chlorins				
955.4902	16.18	C_45_H_84_AsO_14_P	1	arsenosugar 955. As-PL982	glycosyl compounds				
		C_48_H_74_O_19_	0	26 structures					
955.5787	12.47	C_54_H_82_O_14_		8-hydroxy-2,7-bis(methylene)−4Z-octenyl ester and versipelostatin E		C_49_H_82_N_2_O_16_	5	shengjimycins B2α / B2β / B3	aminoglycosides
995.6099	13.84					C_43_H_78_N_16_O_11_	1	K-582-A	oligopeptides
						C_47_H_82_N_10_O_13_	4	aspereline G	oligopeptides

## Bioactivity of pheophorbide a on 2-NBDG assay

**Fig. 7:**
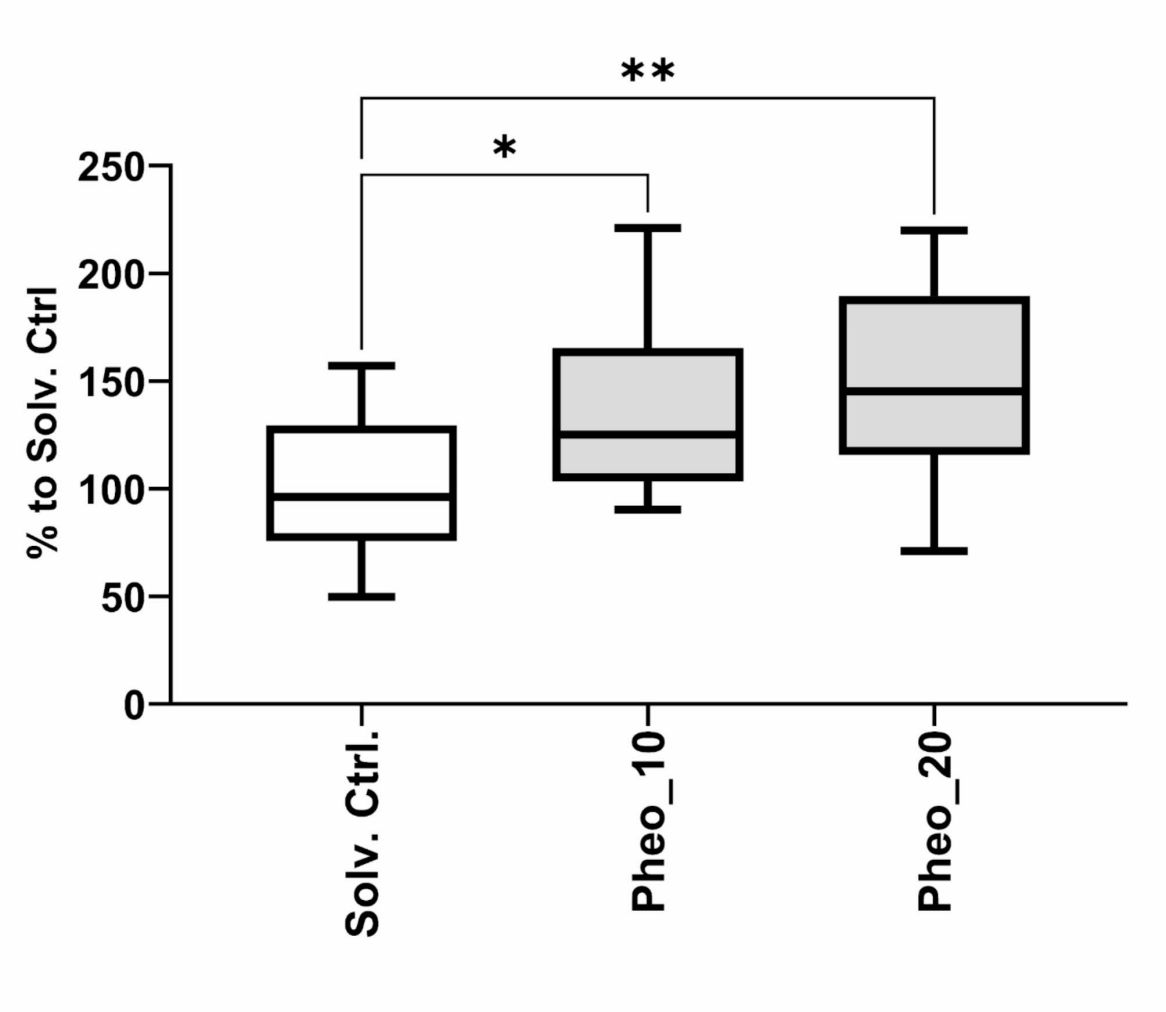
2-NBDG uptake on zebrafish larvae exposed to pheophorbide a, a member of the chlorophyll derivatives. Zebrafish larvae were exposed to pheophorbide a at 10 µM and 20 µM for 1 h, and 2 independent assays were done for each sample (*n* = 16). The data is represented as box-whisker plots from the fifth to 95th percentiles. Asterisks highlight significant differences vs. the solvent control, DMSO 0.1% (** *p* < 0.01; * *p* < 0.05), One-Way ANOVA and Dunnett’s posthoc test.

As the main components of the bioactive fraction 06104_D appear to be chlorophyll derivatives (e.g. 2-Chloro-10*S*-hydroxyphaeophytin a, 13^2^-hydroxy-20-chloro-ethylpheophorbide a derivatives and pheophorbide a derivatives), pheophorbide a (Pheo), commercially acquired, was tested in two concentrations in the 2-NBDG assay. Pheophorbide a significantly increased the 2-NBDG uptake by zebrafish larvae at 10 and 20 µM after 1 h of exposure, as shown on Fig. 7. This finding sheds light on a possible cause-effect relationship between chlorophyll derivatives and glucose uptake, as observed for pheophorbide a and fraction 06104_D.

## Discussion

This work showed the bioactive potential of cyanobacterial strains for the treatment of diabetes. One of the main consequences of diabetes is the increase of glucose levels in the blood, which are regulated by production of insulin to induce the glucose uptake into tissues from the bloodstream^20^. The results of this work revealed two promising fractions (06104_D and 03283_B) that act as insulin mimetics at 10 µg/ml. Their activity was confirmed by two different methodologies, 2-NBDG assay and glucose measurement in whole zebrafish larvae in vivo. To the best of our knowledge, the 2-NBDG assay in zebrafish larvae was applied here for the first time to screen a natural products library. Bioactivity screening with aqueous extracts of two plants, *Tecoma stans* and *Teucrium cubense* (at 70 µg/mL) were reported to increase the uptake of 2-NBDG on insulin resistant adipocytes at 31–94%, and on insulin sensitive adipocytes at 54–193% ^22^. The 2-NBDG assay was applied to zebrafish larvae to study the antidiabetic effect of ginsenoside Rb1, isolated from the american ginseng *Panax quinquefolius*L. This glycosylated triterpenoid increased the glucose uptake by 1.5-fold at a concentration of 20 µg/mL after 1 h exposure^22^.

The measurement of free glucose levels in zebrafish embryos, after exposure to 10 µg/ml of fraction 06104_D and to 20 µg/ml of fraction 03283_B, revealed lower glucose levels for both fractions. This finding is consistent with the 2-NBDG screening results and suggests that the cells of the zebrafish larvae increased their uptake of external glucose, probably related with an increase of consumption^23^. This relationship between glucose uptake and consumption has previously been described in studies involving natural products. The protoalkaloid ρ-synephrine, isolated from bitter orange and other citrus, showed a reduction of glucose in the medium of L6 skeletal muscle cells at a concentration of 50 µM, which was followed by an increase on lactate by the cells, in consequence of glycolysis activation and consequent consumption^24^.

The observed bioactivity for fractions 06104_D and 03283_B can be related to the different mechanisms involved in glucose homeostasis in the distinct organs^25^. An important component of glucose homeostasis are the glucose transporters, commonly known as GLUTs, which are involved in the movement of glucose between different tissues and cells^7^. The distribution of these transporters in body, head and whole larvae demonstrated a significant higher expression of GLUT1 and GLUT3 in the head, compared to the body or whole larvae. These differences are aligned with data from the literature, since GLUT1 and GLUT3 are connected to structures and components of the brain, like the blood-brain barrier and neurons, while GLUT2 is present in organs related to the digestive process (like liver, pancreas or the small intestine)^23^. The analysis of the GLUT 1–3 expression on the zebra fish larvae after exposure to the cyanobacterial fractions 06104_D and 03283_B revealed a decrease in the expression of GLUT1 for emodin and fraction 06104_D, suggesting that the observed increase of glucose uptake in zebrafish larvae is independent of GLUT1. In contrast, a study using a natural product, curcumin, found that at 40 mg/kg, GLUT1 and GLUT3 levels increased in the brains of diabetic rats^26^.

In order to obtain further mechanistic insights, the mRNA expression of genes related to glucose metabolism were analysed. The mRNA expression of *insa*increased significant after exposure to fraction 06104_D, which can be used as a relative quantification of the total levels of insulin in the embryo^27^. Hence, the observed increase of insulin mRNA levels could translate into an increase of uptake of glucose for consumption by the cells of the zebrafish^20^. A lysate of peptides from antarctic krill was shown to increase the expression of insulin gene, at daily administration for 15 days and concentrations from 39 to 156 mg/mL^28^. This is in accordance with our results from the 2-NBDG screening and glucose measurements in the embryo. The increase of insulin production is one of the hallmarks for the diabetes treatment, as shown in^29^, where a library of 4640 drugs was screened at a concentration of 10 µM to search for modulators of insulin expression. 229 stimulators were identified, with some of them exhibiting also an increase of *PEPCK* expression, like pioglitazone, a known drug for diabetes treatment, but also natural products like karanjin, a flavonoid isolated from *Millettia pinnata*^30^.

Taken together, the results from bioactivity screening and biochemical studies showed that fractions 06104_D and 03283_B are promising sources of compounds that can have an effect on different targets connected with the progression of diabetes. These fractions increased 2-NBDG uptake, reduced the levels of circulating glucose and increased the expression of the insulin producing gene, *insa*, one of the main molecules that control the uptake of glucose from the bloodstream in humans.

Following, metabolite profiling was done to analyse the composition of bioactive fractions and the association of compounds/mass peaks to the observed increase in glucose uptake. Two approaches were applied, Classic Molecular Network for searching mass peaks uniquely present in active fractions, and Bioactivity-Based Molecular Network for correlation of mass peaks to the observed bioactivity^31,32^. One important group of compounds was identified in the fraction 06104_D, namely pheophorbide and pheophytin derivatives, molecules that can have a multiplicity of derivatives and different biological applications This group is chemically classified as chlorins, combining porphyrin and pyrrole rings^33^. Pheophorbide a is highly present in photoautotrophic organisms, as in cyanobacteria or microalgae, since its originated from the breakdown of chlorophyll^34,35^. In order to verify if chlorophyll derivatives may be involved in the increased 2-NBDG uptake in zebrafish larvae in our study, we have tested pheophorbide a in the 2-NBDG assay. Indeed, this molecule increased significantly the glucose uptake in the 2-NBDG assay in zebrafish larvae. Considering these results, it seems plausible to assume that some of the masses identified as chlorophyll derivatives were involved in the observed bioactivity. In accordance to our results, pheophytin a isolated from the leaves of *Clerodendrum infortunatum*reduced both glucose and cholesterol levels accompanied by an increase of insulin secretion in rats after administration of 1.25 g/kg of body weight of extracts^36^. Pheophorbide a inhibited advanced glycation end products receptors, molecules with a central role in the development of diabetes, derived from extracts of leaves of *Mallotus japonicus*at a concentration of 0.1 µM^37^. Furthermore, pheophorbide a was shown to modulate glucose concentrations and GLUT1 trafficking in a pancreatic β cell line, INS-1 ^39^.

Another important family identified on the active fractions is the terpenes/terpenoids present on both analysed fractions (e.g. ganuleucoin P and buxmicrophylline F). The putative identified compounds are not known to be produced by cyanobacteria, however there is a vast number of terpenoids produce by cyanobacteria (e.g. hemiterpenes or monoterpenes)^39^. This family of compounds is composed by monomeric isoprene (C5H8) units and, when oxygenated form the terpenoids^40^. Terpenes are known for their beneficial effects on the treatment of diabetes, like the stimulation of insulin release (stevioside), anti-hyperglycemic activity (tinosporaside) or increase of glucose uptake (palbinone) (reviewed in^41^). These compounds can also interfere with key enzymes for diabetes progression and terpenes isolated from essential oils inhibited α-amylase, reducing glucose levels in diabetic conditions at concentrations from 0.39 to 5.50 µmol/cm^−3 43^.

Xanthopylls (e.g. phoenicoxanthin or 4-ketozeinoxanthin), one of the most important groups of carotenoids, are already known for their pharmaceutical and biological potential in different diseases as reviewed in^43^. For example, fucoxanthin, produced by brown algae, increased the expression of uncoupling protein 1 and of GLUT4, reducing glucose levels in rats when exposed at 0.5, 1 and 2% wakame-lipids (lipids with 10% fucoxanthin) in diets^44^. Astaxanthin at 20 mg/kg of body weight ameliorated the condition of alloxan-induced diabetic rats, by reducing hepatoxicity and oxidative stress derived from diabetes^45^.

Lycopene and myxoxanthophyll, carotenoids isolated from *Synechocystis*sp., were shown to inhibit α-glucosidase activity by 43.77% and 46.82%, respectively, being this an enzyme responsible for the uptake of glucose in the intestines, reducing glucose levels in the organism^46^. Spirulina (*Arthospira platensis*), a cyanobacteria already known for its effect on weight loss, showed a reduction in the glucose levels of diabetic rats after administration of the cyanobacteria at 50 mg/kg of body weight, ameliorating the biological conditions caused by diabetes^47,48^.

In summary, this work assessed the bioactive potential of a library of 182 chemical fractions, obtained from 19 cyanobacterial strains. Two fractions (06104_D and 03283_B) showed promising results in the 2-NBDG assay by increasing the uptake of this glucose analogue, and hence acting as insulin mimetics. Both fractions showed a reduction of circulating glucose levels in zebrafish, while fraction 06104_D increased mRNA expression of *insa*, a gene responsible for the expression of insulin. The metabolite profiling of these fractions revealed complex chemical compositions, however, some compounds’ classes could be associated with the observed glucose uptake activity, as the chlorophyll derivatives, xanthophylls and terpenes. The analysis of pheophorbide a as a representative of the chlorophyll derivatives confirmed the increase of the glucose uptake in zebrafish larvae. Future work will be necessary to develop the application of such compounds for the treatment or at least amelioration of the effects of diabetes.

### Methodology

#### Cyanobacteria Growth and Chemical extraction

**Fig. 8:**
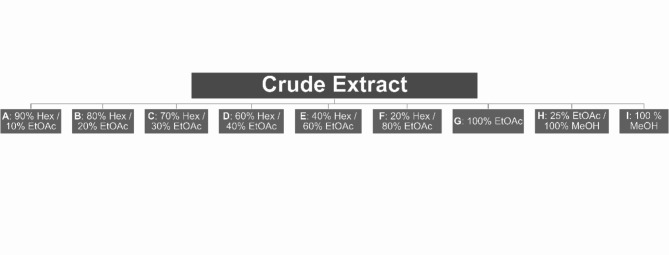
Solvent gradient used for VLC fractionation of crude extracts of the 19 cyanobacteria strains. Solvent proportions are identified by the letters A-I as shown in the figure.

The cyanobacteria growth, harvesting and chemical extraction was done as described in^49^. Briefly, 19 cyanobacterial strains (Table 2), hosted by the Blue Biotechnology and Ecotoxicology Culture Collection (LEGE-CC), were grown under a light/dark cycle of 14/10 h at 25ºC and a photon irradiance of 30 µmol.m^−2^s^−1^, grown in Z8 medium with an extra supplementation for marine strains with 25 g/L NaCl and 20 µg/L vitamin B_12_ (Ramos et al., 2018). Strains indicated on Table 2 were harvested and biomass was freeze-dried. Afterwards, lyophilized biomass was extracted several times by percolation with a warm (≤ 40 ºC) mixture of dichloromethane/methanol (CH_2_Cl_2_:MeOH, 2:1 v/v) (VWR, Radnor, PA, USA), with ultrasound, to obtain organic crude extracts. The extracts were separated by Vacuum Liquid Chromatography (VLC) with a stationary phase of silica gel 60 (0.015–0.040 nm, Merck, KGaA, Darmstadt, Germany) and a mobile phase gradient from 100% n-hexane (VWR) to 100% ethyl acetate (EtOAc) (VWR) and then to 100%methanol (MeOH) (VWR). Figure 8 summarizes the solvents used for VLC fractionation, as an overview. The fractions were dried and resuspended in dimethyl sulfoxide (DMSO) (VWR) at 10 mg/mL and stored at −20 ºC. Table 2 indicates the strain names, identification code, sample locations and habitat. Pheophorbide a (Pheo) was acquired from Santa Cruz Biotechnology (Dallas, TX, USA).

**Table 2:** Strain names, identification code, sample locations and habitat.

Taxon	Strain code	Environment	Fraction Number
*Planktothrix mougeotii*	LEGE 07230	aquatic, freshwater, floating masses, from a secondary decanter tank bank	1–9
*Tychonema* sp.	LEGE 07196	aquatic, freshwater, biofilm, from a biological treatment tank outlet	10–18
*Nodosilinea* sp.	LEGE 06001	aquatic, marine, sea water sample, coastal, surf zone	19–27
*Synechocystis* sp.	LEGE 06079	aquatic, brackish, mesotidal zone, benthic (freshwater)	28–35
*Mycrocistis aeruginosa*	LEGE 91,094	aquatic, freshwater, pond, water sample	36–44
*Nodosilinea nodulosa*	LEGE 07084	aquatic, brackish, mesotidal zone, benthic (freshwater)	45–53
*Nodosilinea nodulosa*	LEGE 06104	Aquatic, marine, tide puddle, rock surface scraping	54–62
*Synechocistis sp.*	LEGE 07211	Aquatic, marine, biofilm, from a biological treatment tank outlet	63–74
Unidentified filamentous Cyanobacterium	LEGE 07212	aquatic, freshwater, biofilm, from a biological treatment tank outlet	75–84
*Tychonema* sp.	LEGE 06363	aquatic, freshwater, biofilm, from a biological treatment tank outlet	85–95
*Limnothrix sp.*	LEGE 00237	Aquatic, freshwater, water sample	96–105
*Nodosilinea sp.*	LEGE 03283	Aquatic, freshwater, water sample, dam reservoir	106–115
*Chroococcidiopsis sp.*	LEGE 06174	Aquatic, marine, sea water sample, coastal, surf zone	116–125
*Cyanobium sp.*	LEGE 07175	Aquatic, marine, sea water sample, coastal, surf zone	126–137
Unidentified filamentous *Synechococcales*	LEGE 07075	Aquatic, brackish, mesotidal zone, benthic	138–146
*Synechococcus sp.*	LEGE 11,428	Aquatic, marine, subtidal sample, epilithic (13 m depth), about 200 m off the shore	147–155
Unidentified filamentous cyanobacterium	LEGE 00060	Aquatic, freshwater	156–164
*Cuspidothrix issatschenkoi*	LEGE 00247	Aquatic, freshwater, water sample	165–173
*Dolichospermum sp.*	LEGE 00246	Aquatic, freshwater, water sample	174–182

### 2-NBDG assay on zebrafish larvae

The evaluation of glucose uptake on Zebrafish was done using a previously described assay with the glucose analogue 2-NBDG (Abcam)^50^. Briefly, 1-day post fertilization (dpf) zebrafish embryos were transferred to E3 medium (NaCl (10mM), KCl (360 µM), CaCl_2_·2H_2_O (660 µM), MgCl_2_·6H_2_O (802 µM) and 1% Methylene Blue in water) with 200 µM 1-phenyl-2-thiourea (PTU) (VWR) to avoid pigmentation. At 3 dpf embryos were exposed to the cyanobacterial fractions at a concentration of 10 µg/mL in a 96-well plate with 5 larvae per well (*n*= 5). DMSO (0.1%) was used as solvent control and emodin (10 µM) (Target Mol, Wellesley, MA, USA) as positive control. After one hour of exposure, medium was removed and replaced with 2-NBDG at 200 µM in E3 with PTU, as previously described. 3 h afterwards larvae were washed, in E3 without methylene blue, several times to remove non-uptaken 2-NBDG. Larvae were anaesthetized with tricaine (MS-22, 0.03%) for 1 min before imaging on a fluorescent microscope (Olympus BX-41, Olympus, Hamburg, Germany). ImageJ was used to quantify the fluorescence intensity of the eye and yolk sac of each zebrafish embryo^51^. Pheophorbide a was tested following the same protocol, in two independent assays, using two concentrations of 10 µM and 20 µM.

### Glucose measurement

Glucose was measured for the positive hits, fractions 06104_D and 03283_B. Embryos at 4 dpf were transferred to a 48-well plate in 1 mL of E3 medium and exposed to controls and fractions. 8 embryos were used per well in triplicates in two independent assays (*n* = 6). DMSO at 0.1% was the solvent control, Triiodothyronine (T3) at 10 µM was the positive control, and fractions at 10 and 20 µg/mL were used. After 24 h embryos were collected and snap-frozen. Glucose measurement was done with the Glucose Assay Kit (Abcam, Boston, MA, USA) following the manufacturer’s instructions. Briefly, the kit extraction buffer was added to each sample, the larvae were homogenized with a Precellys Evolution Homogenizer (Bertin Technologies, Montigny-le-Bretonneux, France) followed by centrifugation at 16,000 ×g for 3 min. Supernatants were then collected and fluorescence was measured at 520/580 excitation/emission in a BioTek Cytation 5 Image Multimode reader (Agilent, Santa Clara, CA, USA) using 96-well black plates with clear bottom (VWR).

### Glucose transporters (GLUT) phylogenetic analysis

GLUT Sequences from *Homo sapiens*, *Mus Musculus*, *Danio rerio* and *Xenopus laevis* were obtained from NCBI (https://www.ncbi.nlm.nih.gov/) and aligned with the MEGA 7 software^17^. Alignment was done by MUSCLE algorithm using standard definitions, and gap regions (more than 3 sequences with aligned positions) were deleted. Initial tree(s) for the heuristic search were obtained automatically by applying Neighbor-Join and BioNJ algorithms to a matrix of pairwise distances estimated using a JTT model and then selecting the topology with superior log likelihood value. The tree is drawn to scale, with branch lengths measured in the number of substitutions per site. Phylogenetic tree was constructed by maximum likelihood with 1000 bootstraps. The analysis was done by comparing the four main GLUTs with known function in the transport of glucose.

### Protein extraction

Zebrafish embryos at 3 dpf were exposed for one hour to controls, DMSO 0.1% and Emodin at 10 µM, and fractions at 10 µg/ml, as previously described in the 2-NBDG assay. 3 replicates were done per condition, in three independent assays. For the analysis of the expression between head and yolk sac, the head was separated from the body of the embryo using a surgical blade, with 30 embryos per condition, in 3 replicates. Embryos were collected and snap-frozen in liquid nitrogen. RIPA buffer (150 mM NaCl; 5 mM EDTA, pH 8.0; 50 mM Tris, pH 8.0; 1.0% IGEPAL CA-630; 0.5% sodium deoxycholate; 0.1% SDS and water) was added to each sample and protease/phosphatase inhibitor cocktail (Thermo Scientific, Waltham, MA, USA). Samples were then homogenized using the Precellys Evolution Homogenizer (Bertin Technologies), placed on ice for 1 h and then centrifuged at 14,000 rpm for 15 min, at 4 ºC. The supernatant was collected and protein quantified using Pierce™ BCA protein assay kit (Thermo Scientific).

### GLUT expression by western blot

For each sample, a master mix was prepared with NUPAGE™ LDS Sample Buffer, NUPAGE™ Sample reducing agent (Life Technologies, Carlsbad, CA, USA) and protein sample (50 µg) in a total volume of 30 µL. The mix was placed at 97 ºC for 10 min and then loaded to the electrophoresis gel (NuPAGE^®^ Novex^®^ 4–12% Bis-Tris gel (Life Technologies)). As a molecular weight ladder, a mix of MagicMark™ XP Western Standard and SeeBlue Plus2 Pre-Stained protein standard (Thermo Scientific) was used. The gel was run at 170 V for approximately one hour, and then transferred to a PVDF membrane using the iBlot™ 2 system (Thermo Fischer Scientific, Bremen, Germany). Gel transfer quality was then accessed by Ponceau Staining (0.1% Ponceau (Merck, Rahway, NJ, USA)) in 5% acetic acid. For each antibody staining, the same steps were followed: (a) membrane block with 5% Milk (VWR) in PBST (GRiSP, Porto, Portugal) for one hour; (b) wash with PBST and incubation with primary antibody in PBST, following the manufacturer instructions, overnight at 4ºC; (c) wash with PBST and incubation with the secondary antibody, following manufacturer instructions, for one hour at room temperature; (d) wash with PBST and image of the membrane using the Luminescent Image Analyser LAS-4000mini (Fujifilm, Tokyo, Japan); (e) membrane stripping using Restore Plus Western Blot stripping buffer (Life Technologies) followed by several washes with PBST. All these steps were repeated with the same membrane for the housekeeping protein, beta-actin. The following antibodies were used: Anti-GLUT1 (Antibody Verify, Las Vegas, NV, USA), Anti-GLUT2 (Abcam), Anti-GLUT3 (Antibody Verify), β-actin Antibody (Caltag Medsystems Ltd, Buckingham, United Kingdom), Goat Anti-Rabbit IgG (H + L) (peroxidase/HRP conjugated) (Elabscience Biotechnology Inc., Houston, Texas, USA) and Goat Anti-Mouse IgG(H + L) (peroxidase/HRP conjugated) (Elabscience Biotechnology Inc.)

### RNA extraction and cDNA synthesis

Embryos at 3 dpf, in E3 medium, were exposed to fractions and control, with 8 embryos per well and 6 replicates per condition. DMSO (0.1%) was used as solvent control and fractions were tested at 10 µg/mL. 24 h after exposure, embryos were collected, snap frozen and stored at −80 ºC. RNA extraction was done with the RNeasy mini kit (QIAGEN, Hilden, Germany) following the manufacturer protocol, and after sample homogenization with the Precellys Evolution homogenizer. RNA was quantified using DeNovix DS-11 FX (DeNovix, Wilmington, DE, USA) and the RNA integrity was evaluated by the 260/230 nm ratios. Furthermore, RNA quality was also evaluated by electrophoresis on a 1.5% agarose gel with SYBR^®^ Safe staining (Life Technologies).

Synthesis of cDNA was performed using the SensiFAST™ cDNA Synthesis kit (Meridian Bioscience, Cincinnati, OH, USA), following manufacturer’s instructions, and 700 ng of RNA from each sample per 20 µL of total reaction volume.

### Quantitative real-time PCR assay

Real-time qPCR was done for 3 target genes and 2 reference genes. The primer design for *glut2* and *insa*was done as described in^27,52^, respectively. Primer for *pepck*gene was designed by Primer3 software spanning an intron, and amplification efficiency was evaluated by serial dilutions of cDNA. For sample normalization, multiple reference gene approach was followed as described in^53^, using the combination of ribosomal protein L8 (*rpl8*) and ubiquitin-conjugating enzyme E2L3 (*ube2l3*) genes. Table 3 shows primer sequences, annealing temperatures and amplification efficiencies. Primers were acquired from StabVida (Caparica, Portugal).

**Table 3:** Primers sequences, annealing temperature and amplification effiency.

Gene	Sense primer (5’−3’)	Antisense primer (5’−3’)	Annealing Temperature (ºC)	Amplification efficiency (%)
*glut2*	CCACCGAAAACATGGAGGAGTT	TGTCATAACAACTGGGCTCTGTG	57	103
*insa*	AGTGTAAGCACTAACCCAGGCACA	TGCAAAGTCAGCCACCTCAGTTTC	65	101
pepck	CTCCGCTCTCCAAGATAGGG	GGTTACAGGGCCAGTTGTTG	57	111
*rpl8*	CTCCGCCACATTGACTTC	GCCTTCTTGCCACAGTAG	59	91
*ub2l3*	GGTCTGCCTGCCTATCATC	TGTATTCTTCTGCCAGGTCTG	59	99

The qPCR reactions were run on a StepOnePlus™ Real-Time PCR System (Applied Biosystems), using 10 µL for each reaction, containing 5 µL of PowerUp™ SYBR™ Green Master Mix (Applied Biosystems), 200 pM of each primer and 1 µL of a 1:5 diluted cDNA. No template controls were included for each run. The qPCR cycling conditions were: initial enzyme activation at 95 °C for 5 min; followed by 40 cycles at 95 °C denaturation for 20 s, 57–65 °C annealing for 20 s (see Table 2) and 72 °C extension for 20 s. A melting curve from 60 °C to 95 °C (with 0.3 °C increments) for 20 s per step was generated for every run. For each gene, RT-PCR reactions were performed in six replicates, and two independent runs were performed.

### LC-MS/MS and GNPS analyses

To identify the compounds that could be responsible for the observed bioactivity, the LC-HRESI-MS/MS (liquid chromatography-high resolution electrospray ionization tandem mass spectrometry) data was obtained for the positive hits (fractions 06104_D and 03283_B) and for other fractions without activity that were from the same genus and had the same polarity. From each sample, 5 µL (1 mg/mL in MeOH) were injected into an HPLC Vanquish (Thermo Fischer Scientific) using ACE UltraCore 2.5 SuperC18 column (75 × 2.1 mm, ACE, Reading UK), at 40 ºC, with a gradient from 95 to 10% H2O/MeOH/formic acid (95:5:0.1, v/v) to 0.5 to 90% isopropanol/MeOH/formic acid (95:5:0.1, v/v) for 9.5 min, keeping the mixture until 15.5 min before returning to the initial conditions, with a flow rate of 1 ml/min (Ferreira et al., 2021). Analysis was done on an Orbitrap Exploris 120 mass spectrometer (Thermo Fischer Scientific) controlled by Orbitrap Exploris Tune Application 2.0.185.35 and Xcalibur 4.4.16.14. The capillary voltage of the electrospray ionization source (ESI) was set to 3.5 and 2.5 kV for positive and negative modes. The capillary temperature was 350ºC. The sheath gas, auxiliary and sweep gas flow rates were at 50, 10 and 1 (arbitrary unit as provided by the software settings) and 70% tube lens. The resolution of MS scan was 60 000, and data-dependent MS/MS was performed on HCD using nitrogen as gas with collision energy settings of 35 V at 1500 resolution. MS data handling software (Xcalibur QualBrowser software, Thermo Fischer Scientific) was used to search by their m/z value and MS/MS value.

For the construction of the molecular networks the mass data of fraction 57 (06104_D) was compared to fractions 22 (06001_D) and 48 (07084_D), while the mass data of fraction 107 (03283_B) was compared to fractions 20 (06001_B) and 55 (06104_B). Hence, raw data files were converted to mzML format with MSConvert, using the parameters recommended for the Global Natural Products Social Molecular Networking (GNPS)^32^. Two approaches were applied: (1) classical molecular networking and (2) bioactivity-based molecular networking. For 1), converted files were uploaded to the GNPS platform and molecular network was constructed using default settings. For 2) converted files were uploaded to MZmine 2 v2.53 ^56^to generate the quantification file for the analysis by the bioactive molecular network on the Jupyter Notebook^31^as well the quantification file and MS2 spectral summary file for the feature based molecular networking on the GNPS platform, using default settings. ClassyFire^18^ was used for compound class identification. The file uploaded to the bioactive molecular network on the Jupyter Notebook was edited by heading a top row indicating the bioactivity of each sample on the 2-NBDG assay. Cytoscape 3.9.1 ^20^ was used to visualize the molecular network from approach 1 and to combine the output data of jupyter notebook and feature-based molecular network on 2.

Possible adducts for each identified mass were verified manually on the mass spectra, namely for [M + H]^+^, [M + Na]^+^, [M + K]^+^ adducts in positive ion mode. In the next step, the putative identification of the selected masses was performed by searching in Natural Product Atlas (https://www.npatlas.org, accessed on 15/10/2023) and Dictionary of Natural Products (https://dnp.chemnetbase.com, accessed on 15/10/2023) online databases for an accurate molecular weight comparison and identification of compounds (Table 1and Supplementary Tables 1–3), using a mass error value of +/- 5 ppm. Drugs and toxicants were removed from the compounds searched. Compound family for each identification was done by ClassyFire^18^.

### Statistical analysis

The normal (Gaussian) distribution of the data was analysed by Kolmogorov-Smirnov tests. If the data followed a normal distribution, One-way ANOVA was used followed by Dunnett’s multiple comparison tests. If data did not meet normality criteria, Kruskal-Wallis test was used followed by Dunn’s test. Statistical analyses were done on GraphPad Prism (https://www.graphpad.com/, version 10.0.0 for Windows, GraphPad Software, Boston, Massachusetts USA, ).

For the 2-NBDG assay of the exposure to fractions and of the pheophorbide exposure, 10 replicates were used from two independent assays (*n* = 10). The glucose measurement were based on 8 embryos per well, in triplicates, in 2 independent assays (*n* = 6). The GLUT expression by Western Blot was done by each 3 replicates in three independent assays (*n* = 9). The mRNA expression by quantitative real-time PCR had 6 replicates per group.

## Electronic supplementary material

Below is the link to the electronic supplementary material.

Supplementary Material 1

## Data Availability

The datasets generated during and/or analysed during the current study are available from the corresponding author on reasonable request.
