# Supplementary material for: Phenotypic screening in zebrafish larvae identifies promising cyanobacterial strains and pheophorbide a as insulin mimetics
Source: Sci Rep. 2024 Dec 30;14:32142. doi: 10.1038/s41598-024-83986-0 (PMC11685485; doi:10.1038/s41598-024-83986-0)
Supplement: Supplementary file 1 — Supplementary Material 1 [file 41598_2024_83986_MOESM1_ESM.docx]

Supplementary Table 1. Feature based molecular networking with GNPS of fraction 06104_D. Manual annotation of unique mass nodes was retrieved from Natural Product Atlas (NPA) and Dictionary of Natural Products (DNP) databases, based on the precursor ion (m/z) and calculated molecular formula within a mass error of ±5 ppm.

|  | | **Dictionary of Natural Products** | | | | **Natural Product Atlas** | | | |
| --- | --- | --- | --- | --- | --- | --- | --- | --- | --- |
| **m/z** | **Retention Time (min)** | **Molecular Formula** | **Mass error**  **(ppm)** | **Tentative Identification** | **ClassyFire Class Annotation** | **Molecular Formula** | **Mass error**  **(ppm)** | **Tentative Identification** | **ClassyFire Class Annotation** |
| 559.2336 | 14.42 | C_33_H_34_O_8_ | 1 | 6 structures (e.g. cassumunin A and C) |  |  |  |  |  |
| 609.2714 | 11.28 | C_34_H_40_O_10_ | 2 | 14 strucutures |  |  |  |  |  |
|  |  | C_35_H_36_N_4_O_6_ | 0 | 4 structures (10-hydroxypheophorbide a and mesopheophorbide b) | chlorins |  |  |  |  |
| 637.3028 | 11.90 | C_37_H_40_N_4_O_6_ | 0 | ethyl 10-hydroxypheophorbide a and 13^2^-hydroxy-17,17^1^-dimethylpheophorbide a | chlorins | C_37_H_40_N_4_O_6_ | 0 | (13^2^S, 17S, 18S)-13^2^-hydroxy-20-chloro-ethylpheophorbide a | chlorins |
| 643.2317 | 15.09 | C_40_H_34_O_8_ | 2 | 3-[(2,7-dihydroxy-4-methoxy-1-phenanthrenyl)methyl]-2-(4-hydroxy-3-methoxyphenyl)-10-methoxy-2,3,4,5-tetrahydrophenanthro[2,1-b]furan-7-ol | 2-arylbenzofuran flavonoids |  |  |  |  |
| 827.5463 | 14.95 | C_53_H_70_N_4_O_4_ | 2 | 13^2^-oxopyropheophorbide a (phytyl ester) and pyropheophytin b | chlorins |  |  |  |  |
| 885.5531 | 14.20 | C_55_H_72_N_4_O_6_ | 0 | phaeophytin b | chlorins |  |  |  |  |
| 921.5298 | 15.13 | C_45_H_68_N_12_O_9_ | 1 | helicokinin III | peptides |  |  |  |  |
|  |  | C_55_H_73_ClN_4_O_6_ | 0 | 2-chloro-10S-hydroxypheophytin a. 20-chloro-132S-hydroxypheophytin a | chlorins |  |  |  |  |
| 931.5940 | 14.16 | C_57_H_78_N_4_O_7_ | 1 | porphyrinolactone | chlorins |  |  |  |  |
| 941.5273 | 12.03 | C_52_H_76_O_15_ | 1 | carotenoid P 457 and 3-O-[3,6,7-trihydroxy-2-naphthalenylcarbonyl-(→4)-β-D-glucopyranosyl-(1→4)-β-D-glucopyranoside] |  |  |  |  |  |
| 943.4978 | 13.92 | C_55_H_71_ClMgN_4_O_6_ | 1 | 20-chloro-132-hydroxychlorophyll a | Chlorins |  |  |  |  |
| 955.5432 | 11.62 |  |  |  |  | C_46_H_82_O_20_ | 5 | F-19848 A | carbohydrates |
| 955.5787 | 12.48 | C_54_H_82_O_14_ | 0 | 8-hydroxy-2,7-bis(methylene)-4Z-octenyl ester and versipelostatin E. |  | C_49_H_82_N_2_O_16_ | 5 | shengjimycins B2α / B2β / B3 | aminoglycosides |
| 959.5883 | 14.08 |  |  |  |  | C_47_H_78_N_10_O_11_ | 5 | Ll-F07b | depsipeptides |

Supplementary Table 2. Classic GNPS molecular networking of fraction 03283_B. Manual annotation of unique mass nodes was retrieved from Natural Product Atlas (NPA) and Dictionary of Natural Products (DNP) databases, based on the precursor ion (m/z) and calculated molecular formula within a mass error of ±5 ppm.

|  | | **Dictionary of Natural Products** | | | | **Natural Product Atlas** | | | |
| --- | --- | --- | --- | --- | --- | --- | --- | --- | --- |
| **m/z** | **Retention Time (min)** | **Molecular Formula** | **Mass error**  **(ppm)** | **Tentative Identification** | **ClassyFire Class Annotation** | **Molecular Formula** | **Mass error**  **(ppm)** | **Tentative Identification** | **ClassyFire Class Annotation** |
| 299.2005 | 8.67 | C_20_H_26_O_2_ | 2 | 84 structures |  | C_20_H_26_O_2_ | 2 | diterpenoid 6-deoxyaspergiloid C | diterpenoids |
| 365.2474 | 10.77 | C_18_H_37_O_5_P | 4 | (2-hydroxyethyl) phosphonic acid | fatty acid esters | C_18_H_37_O_5_P | 5 | 2-acyloxyethylphosphonate | fatty acid esters |
|  |  | C_25_H_32_O_2_ | 2 | spiculoic acid B | styrenes | C_25_H_32_O_2_ | 2 | 15-dehydro-6-epi-ophiobolin G 14 | sesterpenoids |
| 387.2292 | 10.87 | C_22_H_30_N_2_O_4_ | 2 | 6 structures (e.g. 2-phosphonoethyl 9,12,15-octadecatrienoate) |  |  |  |  |  |
| 455.2555 | 11.79 | C_26_H_34_N_2_O_5_ | 2 | 6 structures (e.g. macrodasines A and D) |  | C_26_H_34_N_2_O_5_ | 2 | calcaripeptide B | depsipeptides |
| 483.3212 | 12.34 | C_29_H_42_N_2_O_4_ | 2 | nigramide K | benzodioxoles |  |  |  |  |
| 519.2982 | 12.28 | C_30_H_38_N_4_O_4_ | 2 | lotusine A and sativanine B | peptides | C_29_H_42_O_8_ | 5 | austalide H acid butyl ester cochlioquinone K lucidenic acid P |  |
| 567.4196 | 13.42 | C_40_H_54_O_2_ | 1 | 28 structures |  | C_40_H_54_O_2_ | 1 | 4-ketozeinoxanthin | xanthophylls |
| 569.3815 | 13.56 |  |  |  |  | C_35_H_52_O_6_ | 5 | integracin C | benzoic acid esters |
| 579.3835 | 12.11 | C_40_H_50_O_3_ | 0 | anhydroamarouciaxanthin B, gelliodenone and pheniconone | prenol lipids |  |  |  |  |
| 581.3992 | 12.14 | C_40_H_52_O_3_ | 0 | 14 structures |  | C_40_H_52_O_3_ | 0 | 2-hydroxytorularhodin phoenicoxanthin | xanthophylls |
| 589.4020 | 13.32 | C_37_H_54_N_2_O_4_ | 2 | buxmicrophylline F | triterpenoids |  |  |  |  |
| 603.3813 | 12.17 | C_36_H_55_ClO_5_ | 0 | cylindrocyclophane C1 | phenols | C_36_H_55_ClO_5_ | 1 | cylindrocyclophane C1 | phenols |
| 621.3920 | 12.11 | C_37_H_52_N_2_O_6_ | 2 | 4 structures |  |  |  |  |  |
| 641.3934 | 14.07 | C_35_H_52_N_4_O_7_ | 3 | guineamide C and 14-O-(N-acetylglucosaminyl)teleocidin A | peptides | C_35_H_52_N_4_O_7_ | 3 | guineamide C and 14-O-(N-acetylglucosaminyl) teleocidin A | peptides |
| 749.6223 | 15.00 | C_53_H_80_O_2_ | 1 | plastoquinone 9 and solanochromene |  |  |  |  |  |
| 803.5833 | 16.40 | C_51_H_78_O_7_ | 0 | 5-Deoxy, 6,7-deepoxy, 6,7,26,27-tetrahydro, 20-pentadecanoyl and Indicanine D |  |  |  |  |  |
| 903.5633 | 14.46 | C_55_H_74_N_4_O_7_ | 0 | bidenphytin B, 3,7,11,15-tetramethyl-2E-hexadecenyl ester and talichlorin A |  |  |  |  |  |

Supplementary Table 3. Feature based molecular networking with GNPS of fraction 03283_B. Manual annotation of unique mass nodes was retrieved from Natural Product Atlas (NPA) and Dictionary of Natural Products (DNP) databases, based on the precursor ion (m/z) and calculated molecular formula within a mass error of ±5 ppm.

|  | | **Dictionary of Natural Products** | | | | **Natural Product Atlas** | | | |
| --- | --- | --- | --- | --- | --- | --- | --- | --- | --- |
| **m/z** | **Retention Time (min)** | **Molecular Formula** | **Mass error**  **(ppm)** | **Tentative Identification** | **ClassyFire Class Annotation** | **Molecular Formula** | **Mass error (ppm)** | **Tentative Identification** | **ClassyFire Class Annotation** |
| 419.3153 | 12.69 | C_26_H_42_O_4_ | 2 | 67 structures |  |  |  |  |  |
| 441.2980 | 12.73 | C_23_H_40_N_2_O_6_ | 4 | N2-(15,16-dihydroxy-9,12-octadecadienoyl) glutamine | aminoacids |  |  |  |  |
| 447.3465 | 12.95 | C_28_H_46_O_4_ | 2 | 168 structures |  | C_28_H_46_O_4_ | 2 | 14 structures (e.g. ananstrep A) |  |
|  |  | C_24_H_42_N_6_O_2_ | 4 | 3 structures (e.g. dehydrocrambescin A) |  |  |  |  |  |
| 455.3129 | 12.81 | C_24_H_42_N_2_O_6_ | 2 | acremolide D | depsipeptides | C_24_H_42_N_2_O_6_ | 2 | acremolide D | depsipeptides |
| 469.3294 | 12.94 | C_25_H_44_N_2_O_6_ | 3 | acremolide C and amphimedoside D | depsipetides | C_25_H_44_N_2_O_6_ | 3 | acremolide C | depsipeptides |
| 569.3815 | 13.48 |  |  |  |  | C_35_H_52_O_6_ | 5 | integracin C | benzoic acid esters |
| 747.4806 | 12.95 |  |  |  |  | C_46_H_66_O_8_ | 4 | (2R,3R,2'S)-2-hydroxymyxol 2'-fucoside and (3S,2′S)-4-ketomyxol 2′-fucoside | xanthophylls |

**Total Images of gels from Figure 4A**

GLUT 1 and β-actin


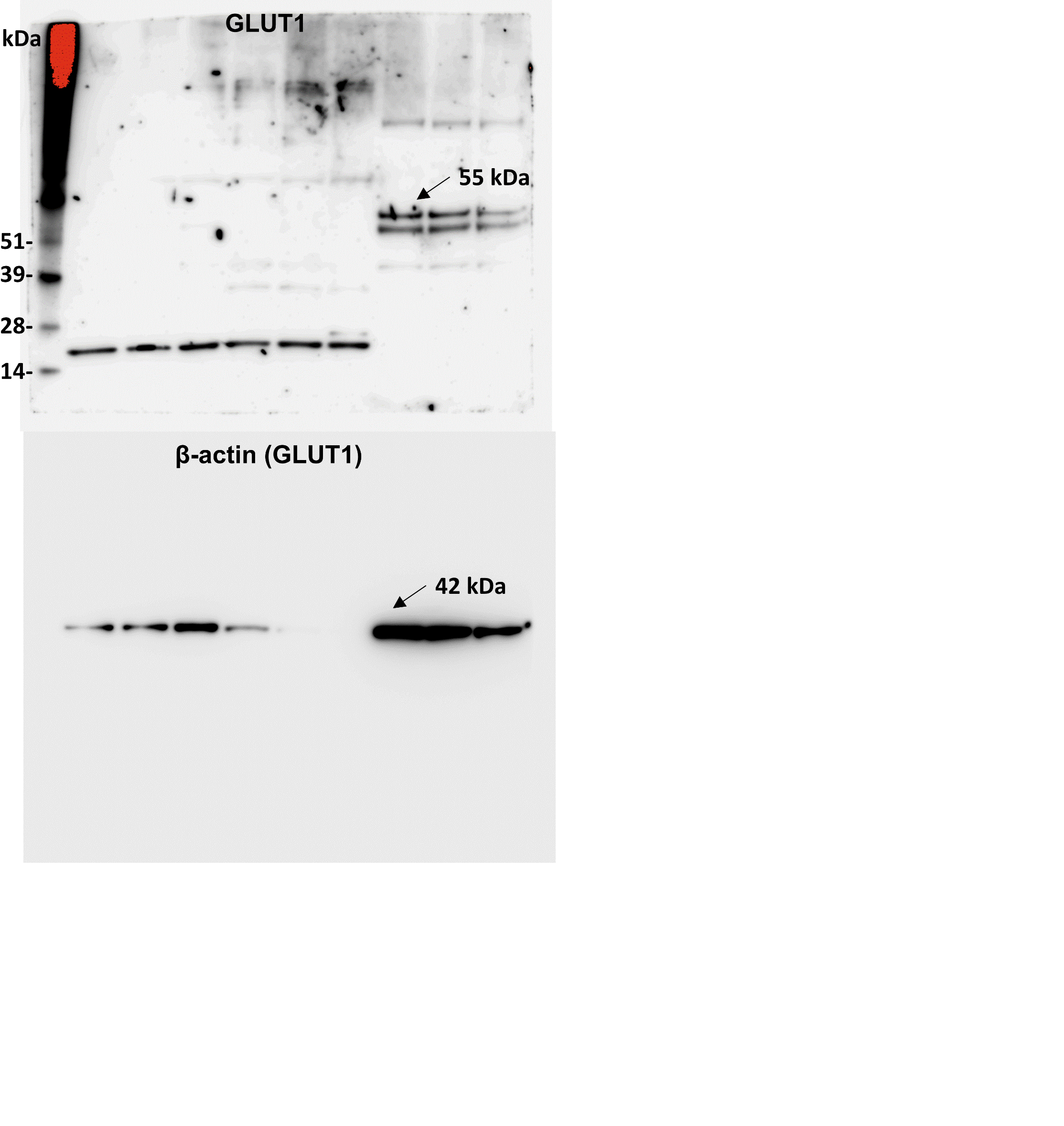


Supplementary Figure 1. GLUT 1 and correspondent β-actin full blot images. Band size for GLUT1 is 55 kDa, while for β-actin is 42 kDa.

GLUT 2 and β-actin


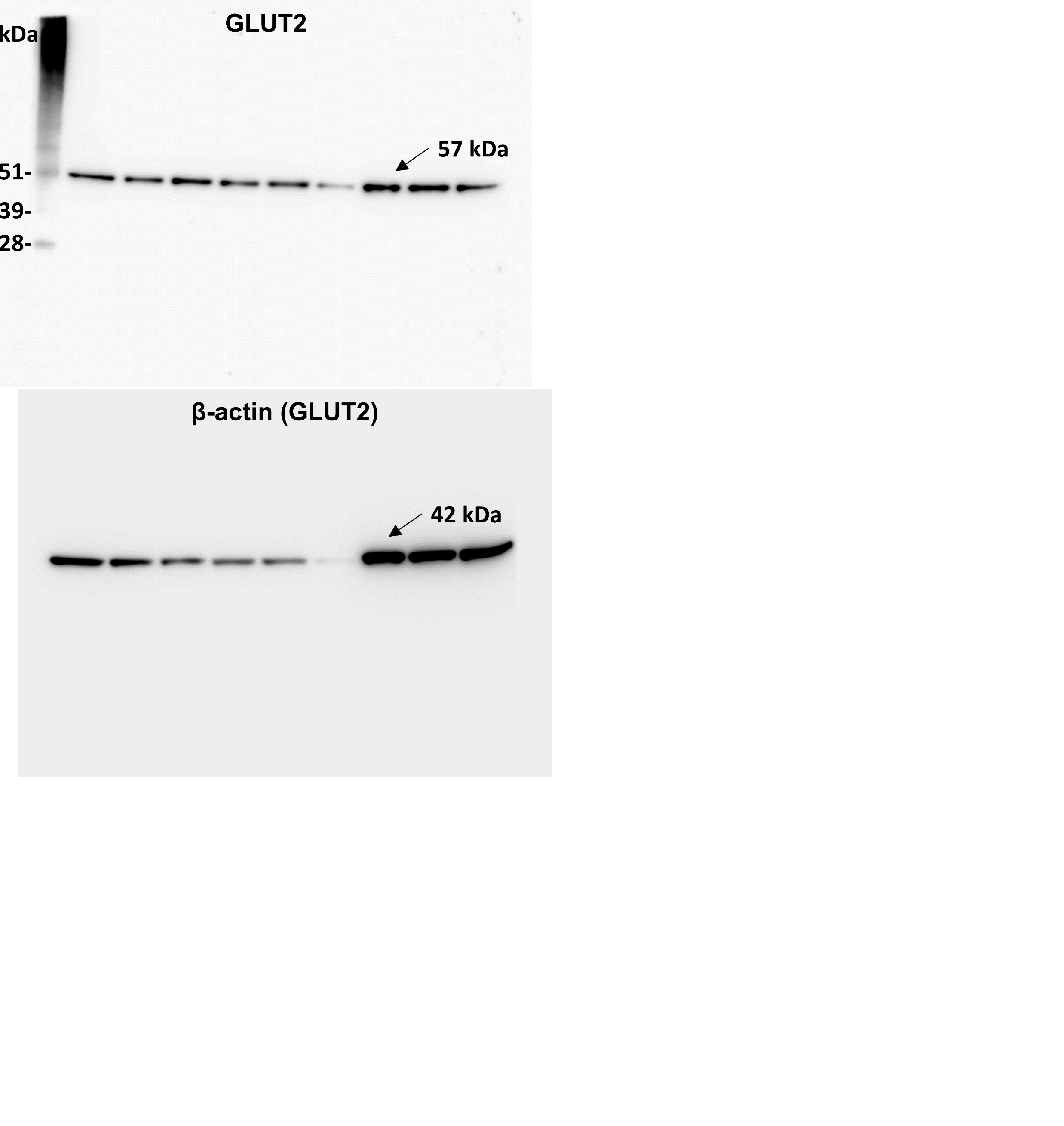


Supplementary Figure 2. GLUT2 and correspondent β-actin full blot images. Band size for GLUT2 is 57 kDa, while for β-actin is 42 kDa.

GLUT 3 and β-actin


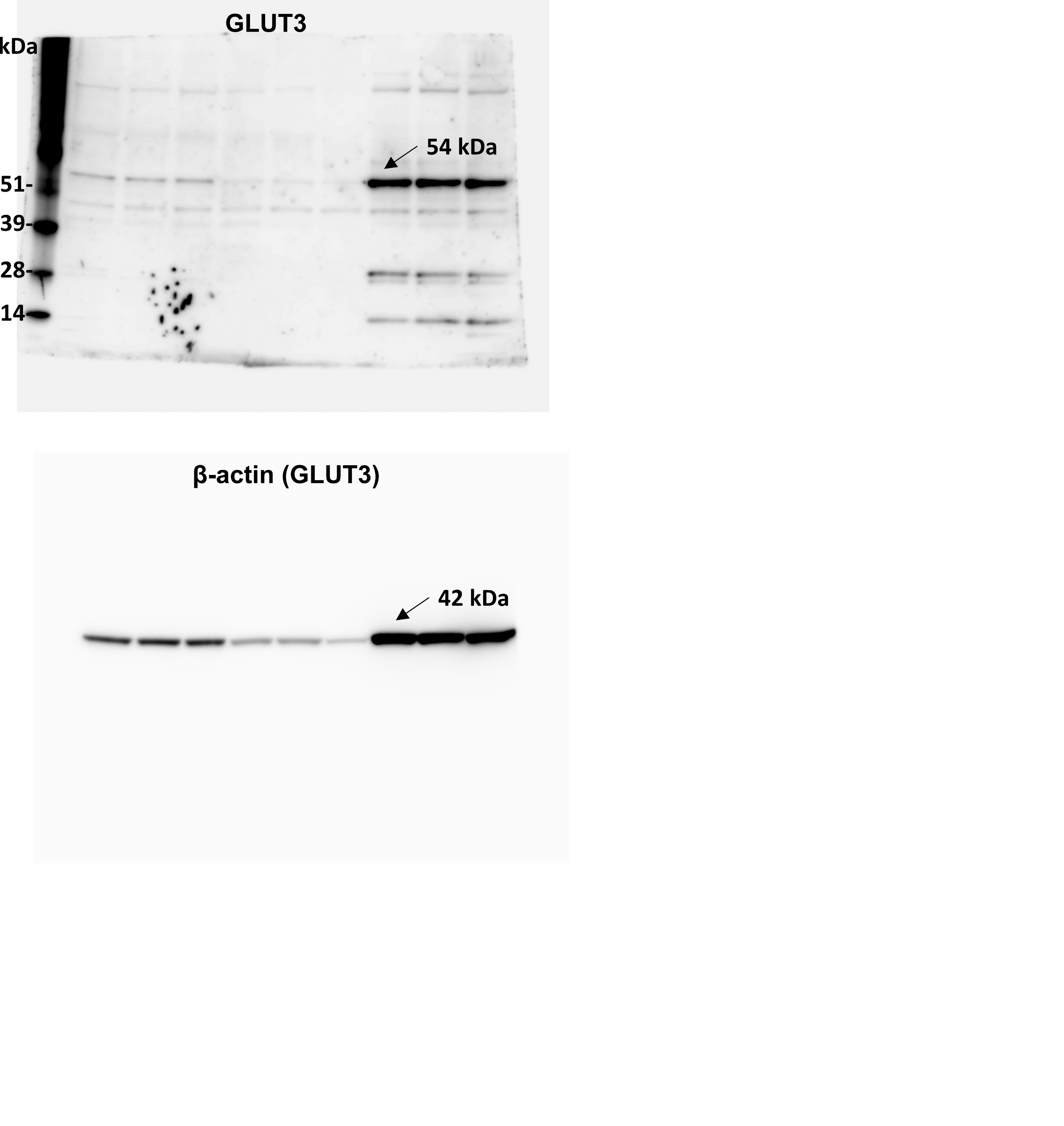


Supplementary Figure 3. GLUT3 and correspondent β-actin full blot images. Band size for GLUT3 is 54 kDa, while for β-actin is 42 kDa.
